# Independent Predictors of 6-Month Mortality in Patients Successfully Resuscitated for Out-of-Hospital Cardiac Arrest: Observational Retrospective Single Center Study

**DOI:** 10.1155/2018/9736763

**Published:** 2018-05-09

**Authors:** Andreja Sinkovič, Andrej Markota, Martin Marinšek, Franc Svenšek

**Affiliations:** ^1^Department of Medical Intensive Care, University Clinical Centre Maribor, Maribor, Slovenia; ^2^Medical Faculty, University of Maribor, Maribor, Slovenia

## Abstract

**Background:**

Mortality of admitted out-of-hospital cardiac arrest (OHCA) patients is decreasing. Our aim was to evaluate independent predictors of six-month mortality of successfully resuscitated OHCA patients.

**Methods:**

We reviewed retrospectively the records of 119 OHCA patients, admitted in 2011 to 2013 (73.1% men, mean age 64 ± 13,5 years) and registered their clinical data, treatments, and predictors of 6-month mortality.

**Results:**

Six-month mortality of admitted OHCA patients was 47.5% and was associated significantly with older age (67.7 ± 12.9 years versus 59.9 ± 13 years, *p* < 0.05), mechanical ventilation, longer time of resuscitation (24.6 ± 18.9 sec versus 8.9 ± 8.4 sec, *p* < 0.05), use of vasopressors (87.3% versus 62.5%, *p* < 0.05), and increased serum lactate (8.1 ± 3.9 mmol/l versus 4.5 ± 3.6 mmol/l, *p* < 0.05) but less likely with prior shockable rhythm (38% versus 73.2%, *p* < 0.05), percutaneous coronary intervention (27% versus 55.4%, *p* < 0.05), achieved target temperatures 32°–34°C of mild therapeutic hypothermia (47.6% versus 71.4%, *p* < 0.05), acute coronary syndromes (31.7% versus 51.8%, *p* < 0.05), and neurological recovery (4.8% versus 69.6%, *p* < 0.05) when compared to survivors. Neurological outcome was most significant early independent predictor of 6-month mortality (OR 50.47; 95% CI 6.74 to 377.68; *p* < 0.001).

**Conclusions:**

Postcardiac arrest brain injury most significantly and independently predicted 6-month mortality in hospitalized OHCA patients.

## 1. Background

During the recent two decades the proportion of patients admitted after out-of-hospital cardiac arrest (OHCA) to ICUs increased significantly due to rapid and effective cardiopulmonary resuscitation provided by emergency medical systems (EMS) [1]. In addition, mortality of admitted OHCA patients decreased significantly, in particular in witnessed cardiac arrests due to shockable rhythms [1]. Nolan et al. demonstrated that the critical care unit mortality for hospitalized OHCA patients decreased from 58.1% in 2004 to 56.9% in 2014 [2]. However, most recent EuReCa ONE study results clearly demonstrated marked differences in reported outcomes following OHCA all over Europe with survival for admitted OHCA patients between 0.2 and 17.3 per 100.000 population per year [3].

Among independent predictors of in-hospital mortality in hospitalized OHCA patients cardiogenic shock and postcardiac arrest brain injury seem the most important [4, 5]. Cardiogenic shock in admitted OHCA patients is the consequence of global hypoperfusion during OHCA, reperfusion injury after return of spontaneous circulation (ROSC), and the trigger of OHCA, being most often acute myocardial ischemia [5]. Postcardiac arrest brain injury is variable, ranging from complete recovery to coma with brain death [4, 6]. It is most widely assessed by cerebral performance category (CPC) scale and corresponds to quality of life and outcome [4, 7].

Since the 2002 mild therapeutic hypothermia with target temperatures of 32°–34°C improved survival and in particular neurological outcome of hospitalized OHCA patients [8, 9], however, recent randomized trial by Nielsen et al. demonstrated that targeted temperature management of 33°C and of 36C° was equal in prevention of postcardiac arrest brain injury for in-hospital outcome in hospitalized OHCA patients [10].

Predictors of longer outcome, such as 6-month mortality, are less well known. Therefore, our aim was to evaluate independent predictors of six-month mortality in admitted OHCA patients.

## 2. Patients and Methods

This was a retrospective, single center observational study, approved by the Institutional Medical Ethics Committee (number KME-05/14). The need for informed consent was waived because of the retrospective nature of the study. Personal data of all the patients were protected according to the law on personal data protection.

We analysed relevant data of OHCA patients, admitted to the Department of Medical Intensive Care (ICU) of the Tertiary University Clinical Centre Maribor, Slovenia, from 2011 to 2013, with the discharge diagnosis “successful resuscitation due to OHCA.” Management of OHCA patients involved mobile EMS, which transferred OHCA patients to the University Clinical Centre, specifically to the Department of Medical ICU only after ROSC was achieved.

750 to 800 admissions per year to the Department of Medical ICU include critically ill medical patients with more than 50% of patients with cardiovascular problems such as ST-elevation myocardial infarctions, acute heart failure, shock syndromes of different origins, postresuscitation patients after in-hospital cardiac arrests and 45–50 successfully resuscitated OHCA patients per year. In addition, patients with severe infections, sepsis and multiorgan failure syndromes, and acute and worsening chronic respiratory failure are admitted as well.

Through the institutional medical information system we obtained the list of consecutive 119 hospitalized OHCA patients (73.1% men, mean age 64 ± 13.5 years) and recorded their clinical, demographic, and mortality data. All the included patients were comatose (Glasgow coma scale – median level 3, mean level 3.6 ± 2.5, range 12) on admission, but with palpable pulse and in majority intubated and mechanically ventilated.

In the time period from 2011 to 2013 approximately 500 OHCA patients were registered, including 300 OHCA patients resuscitated by the EMS and approximately 200 OHCA patients with clear signs of death without resuscitation attempt by EMS. Out of 300 resuscitated OHCA patients 119 were admitted with ROSC (approximately 40% of resuscitated patients) [3].

To overcome the 4 key postresuscitation syndromes such as global systemic hypoperfusion, reperfusion injury, the trigger of cardiac arrest, and the postcardiac arrest brain injury, early postresuscitation treatment goals in hospitalized OHCA patients in ICU were appropriate oxygenation and tissue perfusion, and prevention of recurrent cardiac arrests and of organ dysfunctions [6, 11, 12].

All OHCA patients were noninvasively and invasively monitored by continuous ECG, pulse oxymetry, continuous systemic arterial blood pressure, and central venous pressure measurements [12].

On admission and during the ICU stay blood samples were drawn to measure standard laboratory tests; echocardiography was performed to measure systolic cardiac function, ejection fraction (EF) and left ventricular end-diastolic diameter (LVEDD) [12].

Adequate oxygenation was assured mostly by mechanical ventilation, adequate circulation by infusion of fluids and iv. vasopressors, and/or inotropes and/or mechanical circulatory support by insertion of an intra-aortic balloon pump (IABP) [12]. Postresuscitation care protocol, being in agreement with the guidelines, included oxygenation targets (SatO_2_ 90–95%, arterial pO_2_ 8.5–9 kPa, arterial pCO_2_ 5-6 kPa, and normal pH with serum lactate < 2 mmol/l), ventilation targets (tidal volumes 6–8 ml/ideal body weight), and circulatory targets (mean arterial pressure > 70 mm Hg, heart rate 60–100/min, and ScvO_2_ > 70%) [12].

Convulsions were controlled by anticonvulsant drugs such as benzodiazepines and levetiracetam. Hyperglycemia was controlled by short-acting insulin infusion, accompanied by blood glucose measurements every 2-3 hours with target blood glucose level of 6–10 mmol/l, avoiding hypoglycaemias. Arrhythmias were controlled by antiarrhythmic drugs [12].

In case of ACS, assessed by patients' history, standard ECG recordings, and troponin I measurements, coronary angiography and percutaneous coronary intervention (PCI) were performed [12, 13].

Mild therapeutic hypothermia (targeted temperature management) was initiated as soon as possible after admission to the ICU to prevent postcardiac arrest brain injury [8, 9, 12]. Target core temperature, measured by the urine catheter, was 32–34°C and was achieved by a rapid infusion of 20–30 ml/kg of cold saline of 4°C over 20–30 minutes. The induction time was 60 minutes in average. Mild therapeutic hypothermia was not started in 5 patients, who gained consciousness (Glasgow coma scale 15) soon after admission and in few more patients (5 patients), who died on the day of admission. In addition, in case of worsening respiratory failure and in pulmonary edema the amount of cold saline infusion was reduced, resulting in failure to reach the target temperatures of 32–34°C.

Core temperature of 32–34°C, if reached, was maintained for the next 24 hours by cooled blankets (CritiCool® device, Israel). After 24 hours, gradual rewarming followed in average in the next 6–8 hours until normothermia was achieved [8, 9, 12].

Prognostic assessment of the postcardiac arrest brain injury was started not before 72 hours after admission by neurological clinical examination and if necessary by EEG, CT/MR. Neurological outcome as the consequence of postcardiac arrest brain injury was classified by the cerebral performance category (CPC) scale at ICU discharge [7, 14]. Neurological recovery was defined as CPC 1-2, including minor to intermediate postcardiac arrest brain injury with the ability for independent life. CPC 3–5 defined adverse neurological outcome with severe neurological damage, including vegetative state and brain death [7].

## 3. Statistical Analysis

Statistical analysis was performed using the SPSS® statistical package, version 24 (SPSS Inc., Chicago, IL, USA) for Windows®. Data were expressed as means ± standard deviations or percentages. Differences between the groups were tested by the two-sided Student's* t*-test for means ± standard deviations and by the chi-square test for percentages.

Independent predictors of 6-month mortality of admitted OHCA patients were assessed by the model of binary logistic regression, using Wald statistics.

Kaplan-Meier survival plots were employed to show 6-month survival associated with severe ischemic brain injury (CPC 3–5) and good neurological outcome (CPC scales 1-2).

*p* value of <0.05 was set as the limit of statistical significance.

## 4. Results

Baseline characteristics of admitted OHCA patients are displayed in Table 1. Majority of OHCA patients were men, mean age 64.1 years. Arterial hypertension was the main comorbidity (68.1%), prior myocardial infarction (MI), and diabetes were less likely present. Bystanders resuscitated OHCA patients in 45,4% of cases. Ventricular fibrillation or pulseless ventricular tachycardia (VF/VT) was responsible for OHCA in 54,6%, asystole or pulseless electrical activity (asyst/PEA) in 42%. Mean time from the start of OHCA to arrival of EMS was, when bystander witnessed, in average 8.6 ± 4.5 minutes. The time of resuscitation, performed by EMS to return of the spontaneous circulation (ROSC), was in average 17.6 ± 17 minutes. The cause of OHCA was acute coronary syndromes (ACS) in 41.1% of patients, including evolving acute ST-elevation myocardial infarction (STEMI) in 27.7% and non-ST-elevation myocardial infarction (NSTEMI) in 13.4% of cases.

In-hospital treatments are presented in Table 2.

Majority of OHCA patients were mechanically ventilated; PCI was performed in 40.3%. Mild therapeutic hypothermia was started in majority of patients. Target temperatures 32–34°C were achieved in 58.8% of all OHCA patients. In 30 patients (25.2% of all OHCA patients) temperatures of 34–36°C were achieved; in 19 patients (15.9% of all OHCA patients) temperatures remained >36°C.

Neurological outcome is presented in Table 3. Good neurological outcome classified as CPC scales 1-2 was achieved in 35.3% and adverse outcome, classified as CPC scales 3–5, was observed in 64.7% of admitted OHCA patients.

Six-month mortality of hospitalized OHCA patients was 52.9%.

Comparisons of clinical and laboratory data between hospitalized OHCA patients, who died and who survived within the next 6 months, are presented in Tables 1, 2, and 3. Six-month mortality in comparison to 6-month survival in hospitalized OHCA patients was associated significantly more likely with older age, longer time of resuscitation by EMS, and asyst/PEA, but less likely with male gender and VF/VT, less likely with achieved target temperatures 32–34°C of mild therapeutic hypothermia, less likely with performed PCI, and more likely with vasopressor treatment. Between survivors and nonsurvivors we observed nonsignificant differences in bystander witnessed OHCA, mean time to EMS arrival, and admission EF, in the use of antibiotics, inotropes, diuretics, and IABP (Table 1 and Table 2).

**Figure 1:**
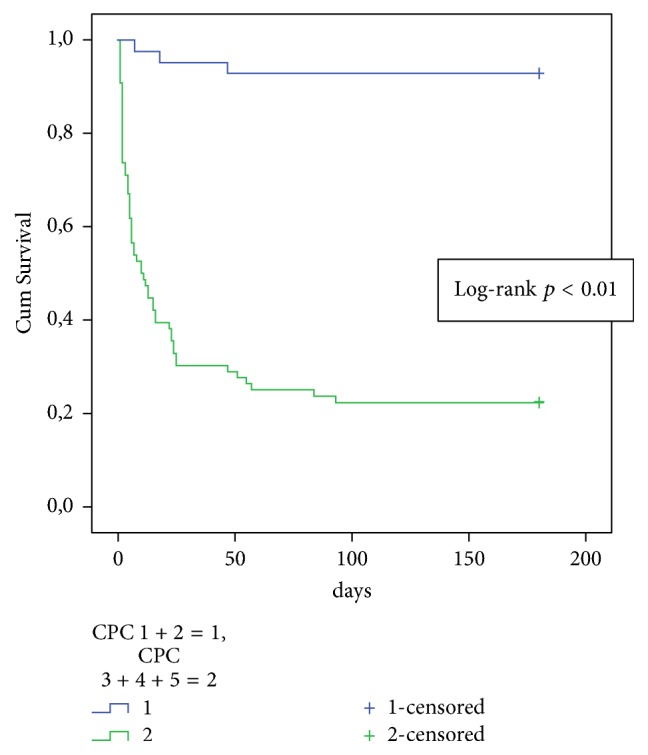
Kaplan-Meier survival curves of hospitalized OHCA patients with CPC 1-2 and CPC 3–5.* Legend*. CPC: cerebral performance category.

Six-month mortality in comparison to 6-month survival in hospitalized OHCA patients was associated significantly less likely with CPC 1-2 and more likely with CPC 3–5 (Table 3). 92.9% of patients with CPC 1-2 survived 6 months and only 22.4% of patients with CPC 3–5 (log-rank *p* < 0,001) (Figure 1).

Neurological outcome, assessed by CPC scale, was most significant independent predictor of 6-month outcome in our hospitalized OHCA patients (Table 4).

## 5. Discussion

In our cohort of hospitalized OHCA patients, 6-month mortality of 47.5% was admitted to ICU. It was associated significantly with older age, longer time of resuscitation by EMS, increased mean admission lactate level, asystole/PEA as a cause of cardiac arrest, mechanical ventilation, and the use of vasopressors, but less likely with the use of PCI and achieved target temperatures 32–34°C of mild therapeutic hypothermia in the postresuscitation period.

Postcardiac arrest brain injury as assessed by CPC scale was even the most significant independent predictor of 6-month mortality of hospitalized OHCA patients.

Postresuscitation period is dominated by a complex process of global ischemia-reperfusion injury and a nonspecific activation of inflammation [6, 11]. The result is depletion of oxygen and energy stores with interrupted cellular functions, with membrane dysfunction and reoxygenation-induced reactions, triggering injury, and toxicity by free-radicals [6]. Pathophysiological background results in particular in severe cardiocirculatory shock, postcardiac arrest brain injury, and other organ failures and inflammation [6, 11].

Postcardiac arrest brain injury is the most devastating postresuscitation syndrome in long-term, depending on the ischemia time and reperfusion period [14]. Global ischemia and reperfusion injury in postresuscitation period cause severely deranged cerebral flow and decreased cerebral oxygen extraction [6, 11, 14]. Therefore, it is not surprising that severe postcardiac arrest brain injury, as assessed by CPC scale, was associated significantly with 6-month mortality of our hospitalized OHCA patients.

Assessment of postcardiac arrest brain injury by CPC scale is simple and widely used in research and in quality assessment following cardiac arrest [7]. The CPC scale at hospital discharge is a useful surrogate measure of long-term survival according to the study by Phelps et al., what was confirmed in our patients [14]. According to postresuscitation guideline from 2015 careful daily clinical neurological evaluation is the basis for proper prognostication in patients after prior successful resuscitation, but it should not be started earlier than after 72 hours of ICU stay or even later in prolonged use of sedatives, neuromuscular blockade, hypotension, hypoglycaemia, and respiratory failure. In any uncertain neurological situation prolonged observation and treatment should be considered. Most survivors recover their consciousness in a week, but some also later. In nonresponding comatose patients several days after admission additional tests should be performed, including brain CT and EEG to prevent the risk of falsely pessimistic prediction [12]. At least one week of ICU-stays is necessary to appropriately predict the brain function after OHCA. In our patients ICU-stay was 9 days in average, even 10 days in survivors and 8 days in nonsurvivors and any withdrawal of treatment was associated with confirmed brain death by clinical examination, EEG, and perfusion brain scintigram.

In addition, heart failure was an important component of outcome in postresuscitation period as demonstrated by studies [5, 6, 11, 15]. In our hospitalized OHCA patients mean admission EF level of included patients was <40%, being nonsignificantly decreased in 6-month nonsurvivors in comparison to survivors. Heart failure was the result of global and also of localized ischemia due to ACS, either STEMI or NSTEMI, which were the cause of cardiac arrest in approximately 40% of patients. 40% of PCIs in our hospitalized OHCA patients corresponded to 40% of ACS in order to overcome myocardial ischemia due to coronary occlusion. According to studies the use of immediate PCI after OHCA in acute coronary events was associated with significantly reduced risk of short- and long-term mortality [16]. Therefore, unified approach with urgent coronary angiography, followed by PCI, was recommended by Radsel and Noc in all OHCA patients with ST-elevation in post-ROSC ECG [17].

In our patiens, mean EF levels in survivors and nonsurvivors were similar in spite of early PCI in STEMI subgroup of patients, as well as the use of inotropes. However, the use of vasopressors was significantly increased in our 6-month nonsurvivors in comparison to survivors, suggesting that shock after ROSC is typically dominated by pathologic vasodilation which persists after normalization of cardiac output. This was demonstrated by others as well [5, 15].

Two landmark clinical trials demonstrated that mild terapeutic hypothermia improved survival and postcardiac arrest brain injury after OHCA [8, 9]. The mechanisms behind are several, including decrease in brain metabolism, reduction of apoptosis and mitochondrial dysfunction, slowing of the cerebral excitatory cascade, decrease of local inflammatory response, reduction of free oxygen radicals, and decrease of vascular and membrane permeability [6, 15, 18]. However, the study by Nielsen and coworkers from 2013 demonstrated that hypothermia not exceeding 36°C was equal to hypothermia of <33°C regarding survival and postcardiac arrest brain injury [10]. In our patients mild therapeutic hypothermia was started in majority of OHCA patients. Reasons not to apply it was most often early death or early improvement (gain of consciousness with Glasgow coma scale 15 soon after admission). However, target temperatures 32–34°C were achieved in 58% of OHCA patients and associated with 6-month survival in our OHCA patients. In additional 30 patients temperatures of 34–36°C were reached only, mostly due to pulmonary edema.

In more than 50% of our patients there was shockable rhythm and in approximately 42% asystole/PEA, but mild therapeutic hypothermia was applied equally in patients with shockable rhythm and in asystole/PEA. A meta-analysis of 15 observational and 2 randomized clinical trials demonstrated a reduction in hospital mortality and improvement in neurological outcome at discharge also in comatose patients resuscitated from non-VF/VT arrest [19].

In one single centre study, conducted by Whittaker et al., four significant predictors of in-hospital death were identified such as nonshockable rhythm, absence of bystander resuscitation, “downtime” > 15 min, and initial pH ≤ 7.11 [20]. In our case series 6-month mortality was also significantly associated with initial rhythm, asystole/PEA and prolonged time to ROSC. In addition, in our patients increased admission lactate was significantly associated with 6-month mortality, suggesting longer ischemia time with the switch to anaerobic metabolism. However, postcardiac arrest brain injury was the most significant independent predictor of long-term mortality in our patients, resuscitated successfully due to OHCA as assessed by logistic regression.

The drawbacks of our study are the retrospective nature, but the strength is the data from the real life. The retrospective data showed that target temperatures 32–34°C were occasionally difficult to achieve by cold saline infusions, as the amount of infused fluids was limited by pulmonary congestion and edema, in particular in ACS patients. Therefore, new methods and devices should be introduced into clinical practice for induction of hypothermia without interfering with respiratory and cardiac function in postresuscitation period. One of them is oesophageal heat transfer device, attaining goal temperatures earlier and with substantially decreased volume of ice-cold saline, as demonstrated in a prospective single centre study by Markota et al. [21].

## 6. Conclusions

Our case series suggest that among several factors postcardiac arrest brain injury most significantly and independently predicted mortality within 6 months in hospitalized OHCA patients. Therefore, every effort should be focused to prevent it or to decease it such as education of population for early call for help of EMS and early start of resuscitation by basic life support in out-of-hospital settings. In hospital settings early induction of hypothermia should be strived for in postresuscitation period to decrease the postcardiac arrest brain injury.

## Figures and Tables

**Table 1:** Baseline characteristics of all hospitalized OHCA patients and comparisons between survivors and nonsurvivors within 6 months.

Variables	All (*n* = 119)	6-month survivors (*n* = 56)	6-month nonsurvivors (*n* = 63)	*p*
Men (%)	73.1	82.1	65.1	0.041
Mean age ± SD (years)	64.1 ± 13.5	59.9 ± 13	67.7 ± 12.9	0.001
AH (%)	68.1	60.7	74.6	0.119
Prior MI (%)	21.8	17.9	25.4	0.378
Diabetes (%)	23.5	17.9	28.6	0.198
Bystander resuscitation of OHCA (%)	45.4	48.2	42.9	0.585
VF/VT (%)	54.6	73.2	38	<0.001
Asystole/PEA (%)	42	21.4	60.3	<0.001
Mean time to CPR ± SD (min)	8.6 ± 4.5	5.7 ± 5.2	7.8 ± 5.3	0.082
Mean time of CPR ± SD (min)	17.6 ± 17	8.9 ± 8.4	24.6 ± 18.9	<0.001
Mean admission lactate ± SD (mmo/l)	6.4 ± 4.2	4.5 ± 3.6	8.1 ± 3.9	<0.001
Mean admission EF ± SD (%)	33.7 ± 15	35.8 ± 15.5	31.2 ± 14.2	0.149
Acute coronary syndromes (%)	41.2	51.8	31.7	0.04
STEMI (%)	27.7	35.7	20.6	0.100
NSTEMI (%)	13.4	16.1	11.1	0.592
Mean GCS ± SD	3.6 ± 2.5	4.3 ± 3.5	3.0 ± 0	0.005

AH: arterial hypertension, MI: myocardial infarction, VF: ventricular fibrillation, VT: ventricular tachycardia, PEA: pulseless electrical activity, CPR: cardiopulmonary resuscitation, SD: standard deviation, EF: ejection fraction, STEMI: ST-elevation myocardial infarction, NSTEMI: non-ST-elevation myocardial infarction, and GCS: Glasgow coma scale.

**Table 2:** In-hospital treatments of hospitalized OHCA patients and comparisons between 6-month survivors and nonsurvivors.

Variables	All (*n* = 119)	6-month survivors (*n* = 56)	6-month nonsurvivors (*n* = 63)	*p*
MV (%)	95	89.3	100	0.029
Achieved temperature 32–34°C (%)	58.8	71.4	47.6	0.015
PCI (%)	40.3	55.4	27	0.003
Vasopressors (%)	75.6	62.5	87.3	0.002
Inotropes (%)	49.6	46.4	52.4	0.583
IABP (%)	6.7	5.4	7.9	0.721
Antibiotics (%)	85.7	89.3	82.5	0.432
Diuretics (%)	46.2	53.6	39.7	0.144
Days in ICU (mean ± SD)	9.2 ± 9.3	10.6 ± 9.9	8.0 ± 9.3	0.134
Days in hospital (mean ± SD)	23.5 ± 27.3	33.2 ± 28	15.4 ± 23.7	<0.001

MV: mechanical ventilation, PCI: percutaneous coronary intervention, IABP; intra-aortic ballon pump, and ICU: intensive care unit.

**Table 3:** Neurological outcome of all hospitalized OHCA patients and comparisons between 6-month survivors and nonsurvivors.

Neurological outcome	All (*n* = 119)	6-month survivors (*n* = 56)	6-month nonsurvivors (*n* = 63)	*p*
CPC 1-2 (%)	35.3	69.6	4.8	<0.001
CPC 3–5 (%)	64.7	30.4	95.2	<0.001

CPC: cerebral performance category.

**Table 4:** Multivariate logistic regression model to evaluate independent predictors of 6-month mortality in hospitalized OHCA patients.

	Adjusted OR (95% CI)	*p*
Target temperatures 32°C–36°C	0.34 (0.75 to 1.54)	0.160
Gender	2.42 (0.52 to 11.15)	0.258
VT/VF/asystole/PEA	1.40 (0.33 to 5.88)	0.647
PCI	0.49 (0.06 to 4.14)	0.514
Mechanical ventilation	5.01 (0.23 to 108.31)	0.304
ACS	0.67 (0.10 to 4.61)	0.687
Post-cardiac arrest brain injury	50.47 (6.74 to 377.68)	<0.001
Vasopressor	3.69 (0.61 to 22.31)	0.155
Age (per 10 years)	1.56 (0.92 to 2.63)	0.097
Admission Glasgow coma scale (per 5)	7.12 (0.67 to 75.63)	0.186
Admission lactate (per 5 mmol/l)	2.04 (0.85 to 4.93)	0.112

VF: ventricular fibrillation, VT: ventricular tachycardia, PEA: pulseless electrical activity, PCI: percutaneous coronary intervention, and ACS: acute coronary syndromes.
